# “It has to be part of the clinic’s lived culture”- perspectives of next of kin on meaningful participation in psychiatric care: a participatory thematic analysis

**DOI:** 10.1186/s12888-026-08561-5

**Published:** 2026-08-26

**Authors:** Nora Dietrich, Johanna Leona Kummetat, Silvia Bahl, Sebastian Bayer, Kolja Heumann, Susanne Kappesser, Thomas Klatt, Sarah Schernau, Sven Speerforck, Sebastian von Peter, Laura Galbusera

**Affiliations:** 1 Department of Psychiatry and Psychotherapy, Brandenburg Medical School (MHB), Rüdersdorf bei Berlin, Germany; 2 Department of Psychiatry and Psychotherapy, Medical Faculty, University of Leipzig, Leipzig, Germany; 3 Institut für Sozialpsychiatrie Mecklenburg-Vorpommern, University of Greifswald, Rostock, Germany

**Keywords:** Next of kin participation, Next of kin perspectives, Informal caregiver, Social psychiatry, Participatory research, Reflexive thematic analysis

## Abstract

**Background:**

The participation of next of kin in psychiatric care is widely recommended in clinical guidelines and research, as it has been associated with improved recovery outcomes and benefits for the wellbeing of next of kin themselves. However, many next of kin report limited opportunities for meaningful participation. Existing research has largely focused on barriers especially from the perspective of clinical professionals, while the perspectives of next of kin regarding what constitutes successful participation remain underexplored.

**Methods:**

This participatory qualitative study was conducted as part of the „PazAng“ research project in Germany. Semi-structured interviews were conducted with 15 next of kin of persons in psychiatric treatment, followed by two focus groups with an additional 15 participants to deepen the findings (total *N =* 30). Data were analyzed using Reflexive Thematic Analysis. Data collection, coding and theme development were conducted collaboratively by a multidisciplinary research team including researchers with and without lived experience as next of kin.

**Results:**

Three main themes were analyzed. First, participants emphasized the importance of a „network-conscious clinical culture“, in which next of kin are recognized as legitimate partners within the social network surrounding the person in treatment and proactively engaged by clinical staff. Second, participants described „different foci of participation“, including interventions supporting the person in treatment, relationship-focused formats, and support specifically addressing the wellbeing of next of kin. Third, participants highlighted „changing needs over time“, describing distinct phases such as an initial shock phase requiring orientation and support, a prolonged endurance phase, and critical transition periods such as discharge from inpatient care.

**Conclusions:**

From the perspective of next of kin, meaningful participation in psychiatric care requires a supportive clinical culture that recognizes relational networks and changing needs over time. The findings suggest that participation is experienced as more meaningful when next of kin are acknowledged as partners, offered different forms of support, and considered across different phases of care. These findings highlight the importance of culturally and structurally integrating next-of-kin participation in routine psychiatric care.

**Clinical trial number:**

Not applicable.

**Supplementary Information:**

The online version contains supplementary material available at 10.1186/s12888-026-08561-5.

## Introduction

The participation of next of kin in psychiatric care can be regarded as a core component of a socially grounded approach to mental health. Drawing on the levels of participation model by Wright, Block and von Unger [1], participation is understood as a continuum ranging from information and consultation to active involvement and shared influence. In the following, we conceptualize participation as distinct from involvement and engagement, emphasizing the opportunities and capacity of those concerned to contribute to, influence, and shape these processes [2]. A substantial body of research demonstrates the effectiveness of next of kin participation in psychiatric treatment. Early and meaningful participation has been associated with improved recovery trajectories of the person in treatment, reduced relapse rates, and enhanced continuity of care [3–6]. In addition, participation in treatment processes has been shown to positively influence the wellbeing, coping capacities, and perceived burden of next of kin themselves [7, 8]. These findings have led to the firm integration of next of kin participation into numerous clinical guidelines and recommendations [9–11]. Moreover, the inclusion of next of kin aligns with the principles articulated in the United Nations Convention on the Rights of Persons with Disabilities [12]. From both ethical and economic perspectives, the participation of next of kin is therefore widely advocated in the scientific literature [13]. Despite these broad recommendations and normative commitments, routine psychiatric care often falls short of this ideal [13]. Many next of kin report dissatisfaction with the extent and quality of their participation, describing experiences of exclusion, insufficient information, and limited opportunities to contribute their knowledge and perspectives [14–19]. A considerable number of studies have investigated barriers to next-of-kin participation, identifying structural, legal, organizational, and attitudinal obstacles [13]. However, these studies have predominantly focused on the perspectives of psychiatric professionals and have frequently been situated within the British healthcare context [13, 20]. To our knowledge, facilitating factors have been examined far less extensively, and even more rarely from the viewpoint of next of kin themselves. Existing research suggests that facilitating factors for next-of-kin participation include professionals’ recognition of relatives as valuable partners, organizational conditions that support family involvement, such as staff training, leadership commitment, and established routines for collaboration [13, 21–23].

Notably underrepresented in the existing research literature is a systematic exploration of what next of kin actually wish for and how they themselves define successful participation. Participation in psychiatric care can take diverse forms, ranging from information sharing and psychoeducation to shared decision-making and collaborative crisis planning [24]. The lack of in-depth knowledge about next of kin’s own perspectives constitutes a significant research gap. A more nuanced understanding of their expectations, needs, and perceived facilitators of successful participation may contribute to the development of psychiatric services that are not only guideline-conform but also responsive to lived experience. Such knowledge is essential for moving beyond a merely formal or tokenistic inclusion of next of kin toward forms of participation that are experienced as supportive, respectful, and genuinely collaborative [2, 25].

Against this background, the present study seeks to explore what constitutes successful participation of next of kin in psychiatric settings from the perspective of next of kin themselves. It aspires to inform the development of psychiatric practices that acknowledge the fundamentally social character of mental health and foster collaborative, supportive, and relational forms of participation.

## Methods

### Design

This study is part of the participatory-collaborative research project “PazAng: Opportunities and barriers to the systematic participation of next of kin in the psychiatric treatment system in Germany”, conducted in cooperation between the Brandenburg Medical School, Leipzig University Hospital, and the Berlin Association of Relatives and Friends of People with Mental Crises (ApK – LV Berlin e.V.), and funded by the German Research Foundation (DFG). The project follows a participatory research approach, involving a multidisciplinary team with diverse professional and experiential backgrounds, including researchers with (“peer researchers”) and without lived experience as next of kin [14]. Formal academic qualifications were not a prerequisite for peer researchers; instead, substantial experience in self-advocacy and self-help was emphasized in order to draw on collective experiential knowledge. A steering board further supported the research process.

### Wording

Key terms were collaboratively discussed and defined within the research team and steering board. We use *next of kin* to refer to individuals in a close and trusting relationship with a person in psychiatric treatment, following a broad understanding of kinship inspired by Haraway [26]. This includes not only biological or legal relatives, but also other significant support persons, such as partners, friends, or roommates, who are involved in the person’s social support network. The term *person in psychiatric treatment* is used to highlight the specific situation that defines the relationship between the three key stakeholders: psychiatric staff, the person in treatment, and next of kin.

A more detailed discussion of terminology and underlying considerations is provided in [14]. The study focuses on adults, given the distinct characteristics of child and adolescent psychiatry.

### Participants

The study draws on two data sources: Firstly, fifteen semi-structured interviews were conducted with next of kin. To further deepen the findings, two additional focus groups were held with a total of fifteen next of kin (total *n* = 30).

Interview participants were recruited via notice boards in clinical settings in Brandenburg and Leipzig, referrals by clinical staff, and self-help organizations. The clinical settings included psychiatric departments within general hospitals, where patients receive inpatient and/or outpatient psychiatric care. Focus group participants were recruited Germany-wide through self-help networks.

Sampling followed a maximum variation strategy to capture diverse perspectives (e.g., treatment settings, kinship relations, gender, age, and duration of involvement). The criteria for the maximum variation sampling strategy were developed and agreed upon through extensive discussions within the participatory research team and the steering committee. Inclusion criteria were age ≥ 18 years and sufficient German language skills. Dementia in the affected person was an exclusion criterion. This decision was based on practical counseling experience, which suggests that relatives of individuals with dementia often assume a distinct caregiving role that is more physically oriented and typically less associated with stigma.

The sample size was guided by the aim of in-depth qualitative exploration and the concept of information power [27]. Further details on recruitment and sampling procedures are reported in [14].

### Data Collection

A semi-structured interview guide (see Additional file 1) was developed through a participatory and iterative process involving several workshops over approximately six months, following the SPSS principle as outlined by Helfferich (2011) [28] (Sammeln, Prüfen, Sortieren und Subsumieren [collecting, checking, sorting, and summarizing]), a structured approach to developing qualitative interview guides. The process began with an initial workshop involving the full research team, focusing on methodological reflection and the collection of relevant topics and questions. Subsequently, smaller groups consisting of researchers with and without lived experience as next of kin and representatives of next-of-kin organizations reviewed and structured the collected material. The results were then discussed in the larger group, further refined in a smaller group through summarizing and synthesis, and finally discussed within the steering group before LG and ND finalized the interview guide.

The guide was designed to ensure that interviews covered specific thematic areas while allowing for flexible, in-depth exploration. The thematic areas included subjective (role) experiences with participation, structural and personal barriers, needs and requirements, and the perceived meaning and benefits of participation. Each area was introduced with an open-ended question and explored further as needed.

Interviews were conducted in person by various members of the collaborative PazAng team. Interview quality was ensured through a team workshop on interviewing techniques. Interviews were held at locations chosen by participants, including self-help organization facilities, clinical settings, or participants’ homes and lasted approximately 60 to 90 min. Participants received compensation of 30 euros for their time.

Following an initial analysis of the interviews, two focus groups were planned as an additional, iterative step of the study design. The focus groups were conducted to further deepen and enrich the findings, particularly with regard to participants’ needs and requirements concerning participation in psychiatric care. They were therefore not part of the initial interview phase but were added after the first analysis. One focus group was conducted in person in Berlin at a self-help organization for next of kin, while the other was held online. Each focus group was facilitated by two researchers with psychotherapeutic or psychiatric professional backgrounds. At the outset, participants were asked to describe what they understood by an ideal form of participation for relatives in psychiatric care. The focus groups lasted approximately two hours, including a ten-minute break after one hour. Participants received compensation of 30 euros for their time.

Following the interviews and focus groups, participants completed a sociodemographic questionnaire, a pseudonymization code, and a contact form, and provided their details for reimbursement. Additionally, interviewers documented their immediate impressions and any noteworthy occurrences on a protocol sheet. The interviews and group discussions were audio-recorded and subsequently transcribed for analysis.

### Analysis

The interview data were coded and managed using MAXQDA [29]. Analytical procedures adhered to the principles of Reflexive Thematic Analysis (RTA) as articulated by Braun and Clarke (2019) [30], which conceptualizes theme development as a creative, interpretive, and reflexive process. This approach is situated within a Big Q qualitative paradigm [31] and is informed by social constructionist and critical realist assumptions, viewing data not as objective reflections of reality but as contextually situated and socially co-constructed narratives.

#### Participatory and reflexive analysis

Consistent with the participatory nature of the research project, analysis commenced with a methodological workshop on Reflexive Thematic Analysis. Steps 1 and 2 (data familiarization and initial coding) were conducted collaboratively by the multidisciplinary team with diverse disciplinary backgrounds (e.g., psychology, cultural studies, and experiential knowledge derived from self-advocacy and self-help) and varying lived experiences as next of kin (e.g., sibling, partner, friend, or none). Team members (SB, SK, SBe, ND) met weekly in a research workshop format, which facilitated communicative validation and iterative reflection.

Theme development proceeded inductively and reflexively. The first generation of themes (Step 3) was undertaken by ND, while the review and refinement of themes (Step 4) occurred through multiple participatory workshops involving a broader team (JK, KH, ND, SB, SK, SP, TK). Throughout the process, coding was treated not as a technical or purely descriptive task, but as an interpretive act in which researchers actively shaped and constructed meaning through engagement with the data [32].

#### Integration of interview and focus group data

The analysis followed an iterative qualitative design in which the interview and focus group data were integrated into a shared, overarching coding and thematic framework. The interview analysis had commenced before the focus groups were conducted. Following the focus groups, their transcripts were incorporated into the ongoing analysis. The focus group data were coded using the emerging coding framework from the interviews, while also allowing for the identification of new codes and perspectives that were not captured in the initial analysis. The coding and themes were subsequently reviewed and refined across both data sources, resulting in an overarching analytic framework that integrated insights from the interviews and focus groups.

#### Positionality and reflexivity

The positionality of the research team was explicitly acknowledged and reflected upon at all stages of analysis. In line with the principles of participatory research [33, 34], team diversity was regarded not as a bias to be corrected but as an epistemological resource. Researchers brought varying degrees of proximity to the subject matter; some identified as next of kin, others possessed cumulative experiential knowledge through involvement in next of kin organizations, and others worked as clinical staff. These differences were not neutralized but embraced as enriching the interpretive process. Rather than striving for consensus or definitive codes, team discussions were used to deepen interpretive insight and critically interrogate underlying assumptions. This approach reflects the reflexive ethos of both participatory and reflexive thematic methodologies.

Efforts were made to avoid tokenistic inclusion by promoting equitable participation. Power dynamics within the team were explicitly addressed and negotiated, for example, through shared authorship, collaborative analysis, and designated spaces for critical reflection. Experiential knowledge was sought not for instrumental use but as equally valuable to academic expertise. Reflexivity was therefore understood not as a discrete analytic phase but as an ongoing, layered process of institutional, personal, and epistemic self-interrogation.

#### Translation of interview excerpts

Interview excerpts were translated from German into English after the analysis and solely for the purpose of this publication. The translations were carried out by the first author, a native German speaker with professional proficiency in English, with particular attention to preserving the original meaning, nuance, and context of the participants’ accounts. As returning translated excerpts to participants for verification would have required considerable additional resources, this step was not undertaken. Additionally, some participants did not have sufficient English proficiency to review the translated excerpts. Instead, the translations were reviewed within the research team to ensure consistency and fidelity to the original German transcripts.

#### Relation to previous publication

The interview data analyzed in this study were drawn from the same qualitative interviews reported in the previous PazAng publication [13]. However, the present manuscript addresses a different research question using a new analytical approach. It also incorporates additional focus group data collected after the initial interview analysis to further explore and contextualize participants’ needs and requirements for participation in psychiatric care. Accordingly, this manuscript represents a further phase of the project, providing a distinct and expanded contribution to understanding the participation of next of kin in psychiatric care.

## Results

### Sample

The semi-structured interviews were conducted in Germany between November 2023 and March 2025. To further deepen the interview findings additional in-depth focus groups held in October 2025. Five interviews were conducted by two peer researchers, seven by two academic staff members with a psychotherapeutic background, and three by an academic staff member specializing in medical quality research. Both focus groups were moderated by academic staff members with a psychotherapeutic and psychiatric background.

Within a short period of time, we received numerous requests from next of kin to participate in both interviews and focus groups, particularly from individuals who became aware of the study through next of kin advocacy groups. The number of requests substantially exceeded the number of interviews we were able to conduct (approximately twice as many), which may reflect the relevance of the topic. Interview participants were selected according to our predefined maximum variation sampling criteria, insofar as this information was available in advance.

Participants came from seven different German federal states. Further sociodemographic details of the participants are presented in Table 1.

**Table 1:** Sociodemographic characteristics of N=30 next of kin

			N=15 (interviews)	N=15 (FG)
Characteristic	N (total)	Proportion*	N (interviews)	N (focus groups)
Age				
< 40 years	3	0.10	2	1
40-60 years	11	0.37	5	6
> 60 years	11	0.37	5	6
Not indicated	5	0.17	3	2
Mean (SD)	56.52 (13.08)			
Gender				
Female	23	0.77	11	12
Male	7	0.23	4	3
Other	0	0.00	0	0
Kinship relation (multiple answers possible)				
Mother	11	0.37	5	6
Father	2	0.07	1	1
Sister	3	0.10	2	1
Son	1	0.03	1	0
Daughter	3	0.10	1	2
Partner, female	6	0.20	2	4
Partner, male	4	0.13	2	2
Friend	3	0.10	2	1
Aunt	1	0.03	1	0
Roommate	3	0.10	2	1
Granddaughter	1	0.03	0	1
Sister in law	1	0.03	0	1
Duration of involvement as next of kin				
<5 years	8	0.27	2	6
>5 years	22	0.73	13	9
Mean (SD)	15.30 (10.56)			
Currently living in the same household				
Yes	6	0.20	2	4
Sometimes	6	0.20	2	4
No	18	0.60	11	7
Involvement in a next of kin’s organization (e.g. participation in its activities or active contributaion)				
Yes	24	0.8	10	14
No	6	0.2	5	1
Treatment setting (multiple answers possible)				
Inpatient Treatment	29	0.97	15	14
Day clinic	11	0.37	5	6
Home Treatment	7	0.23	3	4
Outpatient Treatment	24	0.80	10	14
Experiences of coercive treatment	8	0.27	5	3

* Proportions are based on the total number of participants (N = 30). For variables allowing multiple responses or multiple mentions, proportions may not sum to 100%

### Reflexive thematic analysis

From the interview and focus group material, we developed three central themes that shape the needs described by next of kin within the psychiatric care system in our data: “network-conscious clinical culture,” “different foci of next of kin participation“ and “changing needs over time”.

#### Network-conscious clinical culture

In many participants’ accounts, it became evident that next of kin express a central need that relates less to specific interventions and more to the fundamental attitude of clinical staff. This need can be described as a desire for a network-conscious stance among clinicians. It refers to a basic professional attitude in which next of kin are actively acknowledged in their role within the social network surrounding the person in treatment.

##### Recognition of next of kin as important part of the support network

Many next of kin emphasized that they do not wish to be perceived merely as peripheral actors or even as disruptive influences but as integral members of a relevant support network. Several respondents stressed that sustainable support for the person in treatment is only possible through cooperation with this social network.

And to put it very briefly, what I would wish for is basically this … When a person is ill, there are always accompanying persons, that is, a network. In principle, only a network can truly support the person in treatment. (P24, FG2)

It’s simply not enough to officially offer such services. That also has to be lived within the clinic. The staff need to be informed about it. It would have to be … It has to be part of the clinic’s lived culture. (P25, FG2)

Central to the experience of a network-conscious clinical culture for many interviewees was the feeling of being acknowledged and taken seriously. Next of kin expressed the desire for their perspectives, knowledge, care work, as well as their worries and fears, to be seen and validated. Recognition thus referred both to emotional aspects, being understood in the situation, and to the concrete contributions that next of kin provide in the daily lives of persons in treatment.

But at least there should be recognition that next of kin contribute significantly to the lives of persons in treatment. Without them, things would be very different. (I 8)

Recognition, being seen. I think … I would also wish that people would notice what we do, in the way we can and the way we are, as next of kin. (P16, FG1)

##### Proactive engagement and structural participation of next of kin

The interviews underlined that, from the participants’ perspective, such an appreciative image and a corresponding network-oriented attitude among clinicians is particularly reflected in the fact that clinical staff feel responsible for identifying key next of kin early and actively engaging with them.

That you simply check this in the first few weeks: ‘What does your external support system look like? And do we need to integrate it here, or not?’ (I 1)

Next of kin wished for proactive engagement, such as through a low-threshold conversation offer or by being informed about external support services. Even simple gestures of reaching out were described as potentially helpful:

I don’t really know where to start. Something like a friendlier hospital. But what would that be? Would someone have come to me and said, ‘Hello, you’re probably the daughter of this crazy woman. What do you actually need?’ (I 2)

A conversation offer would have actually been great. Like if they had said, ‘If you have time, we have office hours.’ Specifically for the next of kin. (I 6)

At the same time, it was emphasized that the initiative for cooperation should come from the clinical staff, but participation should not occur against the will of the person in treatment. Rather, the initiative should lie with the clinic, clearly presenting the benefits and repeatedly offering opportunities for conversation without exerting pressure:

Making sure that the environment is included as well. I think you can’t expect that from the person in treatment; it has to be initiated by the clinic. And in my experience, if my daughter is asked whether it’s okay for us to have a joint conversation, she also agrees. (I 11)

Institutionalized offerings, such as next of kin offices or telephone consultation hours, which enable easy accessibility, were highlighted as positive examples:

We have what’s called a next of kin office in the clinic. We really have a room there where we can meet with next of kin. … On ward in this clinic, there are two telephone appointments in the afternoons, where the ward doctor basically offers a phone consultation. You can reach them and ask about whatever is needed. (P 26, FG2)

##### Power sharing

Another facet of a network-conscious attitude was described by a focus group participant as “power sharing” (P27, FG2). This referred to a willingness among clinicians to share interpretive authority by acknowledging that professional expertise represents one perspective among others, while also making their own uncertainties, limitations of knowledge, and emotional burdens transparent. Some interviewees described this as conducive to cooperative, triadic collaboration.

Several respondents criticized that clinical communication even in trialogical seetings often remained limited to technical-professional responses, which they perceived as insufficient for a genuine exchange with next of kin:

They always only respond to technical questions, and that isn’t a good group discussion. (I 14)

In contrast, there was a desire for clinical staff to also show themselves as human and to acknowledge their own limits. Revealing feelings of being overwhelmed or frustrated when dealing with severe illness courses was experienced not as a weakness, but as destigmatizing and relationship-building:

A doctor should also sometimes say: ‘…When I’m facing a manic patient, … I sometimes feel helpless or very, very frustrated. ’ … That would be really good. (I 14)

Such statements were described as a way to normalize the emotional experiences of next of kin and to enable joint engagement with uncertainty. “Power sharing” was also described as a structural issue closely linked to stigmatization within clinical settings. As long as professionals do not bring in their own experiences or uncertainties, the hierarchical separation between professional and experiential knowledge remains:

They have to do power sharing, otherwise they can’t empower. They also need to say, where does the knowledge lie? Maybe it actually lies with the persons in treatment themselves and with the next of kin? And how do you understand your own role? And as long as there’s such a strong stigma in the clinic, where no one comes out with their own experiences, you know, their own knowledge as next of kin or from persons in treatment, then the hierarchy can’t be overcome… (P27, FG2)

A next of kin described a positive experience as follows:

What is schizophrenia? Actually, there aren’t really separate schizophrenias; these are all psychotic crises that any of us can experience at some point. He (the doctor) also immediately gave an example from his own family. And so on. I understood it as: Don’t worry too much … It really took a lot of weight off my shoulders. (I 10)

Taken together, these accounts suggest that, from the interviewees’ perspective, power sharing did not imply relinquishing professional expertise, but rather integrating professional knowledge into a triadic exchange in which the knowledge and experiences of persons in treatment and next of kin are also acknowledged.

In summary, the results highlight that, from the participants’ perspective, a network-conscious clinical attitude represents a central prerequisite for satisfactory participation. This attitude was described as involving recognition of the relevance of next of kin and the wider social network within treatment, an appreciative stance towards both persons in treatment and next of kin as holders of valuable knowledge and experience, and a professional responsibility to proactively engage and involve next of kin in clinical processes.

#### Different foci of participation

In the interviews and focus groups, various specific interventions were identified as helpful or desirable. It seemed useful to us to analyze these based on their underlying objectives: First, many interviewees wished for interventions that would enable next of kin to better support the person in treatment in everyday life. Second, there were numerous accounts indicating a desire for relationship-focused formats that emphasize shared experiences. Third, many interviewees also expressed a wish for support offerings that focus on their own well-being.

##### Interventions focusing on the person in treatment

Many next of kin interviewed described a desire for information and exchange with clinical staff in order to support the person in treatment effectively.

So that the family can prepare for it, and so that the family also has the opportunity to find out somewhere how to handle situations with the person, and what options there are to support them so that things get better again. (I 12)

And of course you also want to care and do the best for the child … And I would have simply wished that someone, um, yes, explained the illness to us and also provided behavioral strategies alongside the treatment. (P28, FG2).

Even low-threshold information offerings were experienced as helpful in reducing initial uncertainties and providing access to further information:

An info flyer with something like: ‘Check out depression.de’ or something like that. I think that would have already lowered that first barrier a little. (I 13)

Offerings that provided next of kin with explanations were highlighted as positive, for example by providing a changed perspective on stigmatized illnesses:

The treating doctor already invited us to a kind of training in the first week, where other parents were also present. And explained to us what psychosis is, what schizophrenia is, and what … we could expect. (P26, FG2)

And a completely new perspective on the frightening concept of psychosis, which, on the advice of (Doctor’s Name), I then read extensively about in the literature, and it really resonated with me. (…) But it was a real enrichment for me to learn things and to understand why they are the way they are. And also to know how to approach the problem at all, so that things can get better, right? (I 10)

Other participants emphasized that participation should remain voluntary and responsive to changing individual circumstances. Some described situations in which they no longer wished to receive information, while others stressed the importance of professionals recognizing when next of kin were overwhelmed and unable to provide further support.

I can also say: ‘I actually don’t want to know all of this anymore.’ Okay, that’s completely fine. (I 13)

It should also be acknowledged when someone says: ‘I can’t do this anymore,’ or ‘I don’t want to anymore,’ or ‘I can’t help because I’m overwhelmed myself.‘ (I 13)

Some participants therefore argued that involvement should be offered rather than expected. While appreciating proactive opportunities for participation, they emphasized that next of kin should remain free to decide whether and to what extent they wished to engage.

If it’s offered, then I can decide whether I want to go or not… But if people kept calling me all the time, that would get on my nerves as well. I also want to live my own life. (I 4)

##### Relationship-focused interventions

From various accounts of next of kin, a need becomes evident for interventions that explicitly focus on the relational dynamics between persons in treatment and next of kin, thereby emphasizing the shared experience. In particular, joint, triadic, or network-oriented conversations were described as meaningful because they provide a structured framework to address relationship issues, promote mutual understanding, and strengthen protective aspects of the relationship for both sides. One next of kin described it as follows:

And if I were in therapy, maybe also talking about it together with them, that also helps me, because I know what they think about it, and I can say what I think. And then maybe we can find a common ground … Our relationship level / it might improve there. (I 13)

Some next of kin who had already experienced network- or triadic-based conversations described these as a special space for encounter, which is hardly accessible in everyday life especially during acute crisis episodes. One mother reported that conversations in the clinical setting were possible despite her daughter’s limited ability to concentrate, and that she experienced these conversations as highly relieving. Another mother described it as follows:

We finally managed to talk about things we normally wouldn’t … when I say, ‘Come sit, I want to talk to you about something,’ I can really feel the walls going up. (I 10)

In the best case, the clinic was experienced as a mediating or triangulating instance, enabling contact to be maintained despite high stress, promoting mutual understanding, and facilitating concrete agreements:

…the clinic was always very helpful, sort of as a mediator to bring us together and … to be able to make arrangements. (I 11)

At the same time, these conversations opened up topics that could hardly be addressed in everyday social life. One next of kin described that the formalized framework helped make actual conversations possible.

Alongside these positive experiences, ambivalent aspects of triadic conversations were also discussed. In a focus group, one next of kin described the difficulty of speaking openly with professionals in the presence of the person in treatment without jeopardizing the trust relationship:

As a next of kin, I can’t speak frankly with the doctor without probably hurting the person in treatment … and that puts you in a … really difficult situation. (P26, FG2)

Some participants highlighted that triadic conversations require careful facilitation to ensure that persons in treatment do not experience communication with next of kin as decisions without their participation. Rather than questioning the value of triadic formats, these accounts emphasized the importance of transparency, trust, and careful negotiation of what can be discussed in the presence of all parties involved.

It really requires a very sensitive approach when you’re talking about this three-person constellation. I think it’s very important to prevent the person in treatment from feeling that everything is being decided behind their back together with the next of kin. I think that’s actually fatal … And ultimately not good for the next of kin either. (I 3)

In the focus group, some participants discussed that while triadic formats were generally considered valuable, participating in these conversations required a process of learning how to express concerns openly yet sensitively in the presence of the service user, while at the same time fostering trust and strengthening the relationship. Reflecting on this experience, a participant described this communication process as something that developed over time:

I would also advocate for triadic formats or network conversations. Although I would say that you definitely have to learn it. Of course, I held back and would have spoken differently if I had been alone with the psychologist, but maybe that’s not even good. So maybe it was good that I had to learn to formulate it more cautiously while my sister was present. Also for the trust, so that she could really see what I had to say. (P27, FG2).

##### Interventions focusing on next of kin

Another central area of findings concerns the need for interventions that focus on the well-being of next of kin.

So that I also have the opportunity to say, ‘Okay, I also need support myself now.’ That this is also acknowledged. (I 13)

Several participants reported that low-threshold support offerings explicitly directed at next of kin were experienced as supportive. These included individual conversations that provided space to reflect on one’s own situation:

The peer support worker … took the time to talk with me, to ask, ‘How are you actually doing?’(I 10)

Multiple participants described exchanges with other next of kin, for example in facilitated open group formats, as particularly meaningful and supportive. These were considered a safe space for sharing, emotional relief, and normalization. One person, for example, reported that they were able to attend a group in the hospital every two weeks, where both next of kin and professionals were present.

And you could just … share your story. You always want to be able to tell it without someone staring at you in shock. (I 7)

Additionally, interview participants expressed the desire to be actively informed by clinical staff about self-help offerings. Exchanges with other next of kin provide the feeling of not being alone with one’s own experiences. In self-help groups, it was also experienced as relieving to be able to express feelings unfiltered, without being judged:

You also have to be allowed to say: ‘This is unbearable and I don’t want to do it anymore.’(I 14)

Self-care, setting boundaries. How can I set boundaries without feeling guilty, right? And to say: ‘Yes, I am there, but I also have a right to my own life, to enjoy my life as well.’ (I 4)

#### Changing needs over time

Many accounts from next of kin underlined that their needs in relation to psychiatric care are not perceived as static but change procedurally over time.

So after the shock phase, I was occupied with the shock, right? I lost 13 kilos during that time, which didn’t have to happen. Then I pulled myself together for a year, and then came this transition phase. (P29, FG2)

At 15, 16 years old, I no longer felt the need to talk to doctors. Why? Well, except to ask, ‘Can I do anything?’ But I mean, I already had enough experience myself. So, I think that’s especially at the beginning. (I 14)

Yes. So now I finally know what wishes I have for the clinicians. And I first had to grow into that role. In that sense, it was good that I had positive experiences at the beginning and now know how things could work. I also know how things could work even better and what expectations I might be able to express regarding the treatment. (I 11)

##### Shock Phase: “I was just so shocked, I wished someone would talk to me”

Many next of kin described the onset of a mental health crisis and psychiatric treatment as a “shock” (I 14) with specific needs particularly orientation and support that next of kin have in this initial situation. Entry into the psychiatric care system was described by many respondents as sudden, overwhelming, and existentially unsettling:

But we next of kin are completely clueless. It hits our lives like a bomb, and we know nothing. (I 14)

A central wish in this early phase was to be recognized as next of kin and actively engaged.

So if they say, ‘Yes, Mrs. (Name), it looks like this and that,’ and don’t exclude me … I actually feel completely alone and helpless. You have to imagine, as a mother or as next of kin … You get scared and panic when … your child or partner is doing so badly. (P17, FG1)

A fundamental need identified across several accounts was initial orientation. This included explanations of what a psychiatric crisis can mean, how psychiatry works, and what next steps to expect. Several participants described destigmatizing, normalizing explanations and an initial orientation offer as helpful:

What is actually happening? Because at least my family couldn’t do that. No one knew what a psychosis is. … And someone could have said something like: ‘Hello, this is a psychiatric facility. This is a place where we treat people who suddenly act very differently, or for a longer period of time act very differently, and are not doing well,’ whatever. ‘And that seems to be the case with your mother now. And maybe it would be good if we sit down together in the near future and see how things proceed. But we have good hope. This can happen in life.’ (I 2)

##### “Prolonged Endurance” Phase

Some interviewees described being a next of kin as a kind of “prolonged endurance run” (P29, FG2) which gradually drains emotional, physical, and social resources over the long term:

The longer the illness continues, the more it drains all your strength. And eventually, you have no strength left. (I 12)

In this context, it became clear that some next of kin desire not only occasional support during inpatient stays but also long-term, continuous accompaniment.

Inpatient, outpatient – and then I lacked support during the prolonged endurance phase, the continuous part. And that’s what really drains you. (P29, FG2)

Particularly emphasized was the responsibility of psychiatric facilities to support next of kin not only in acute crises but also over the long term. Some respondents explicitly framed this as a shared responsibility of the treatment systems:

The psychiatric ward has at least a 50% responsibility for the accompanying next of kin, simply to strengthen them for the endurance phase. (P29, FG2)

##### Needs in the transition from inpatient to outpatient care

In the interviews and focus groups, many participating next of kin described the transition from inpatient to outpatient care as a phase with specific support needs. A key aspect highlighted was discharge management that is closely linked to everyday life and actively involves next of kin. Several respondents criticized that discharges often occur at short notice and without sufficient preparation:

Keyword: discharge management. It would already help a lot if the attitude … were like: ‘We involve next of kin. (P30, FG2)

Respondents found it particularly problematic that discharges sometimes occur without information and without clear agreements:

So another difficult point is always the discharge situation or discharge management, because, exactly, you’re basically pushed out, and then, bam, it’s somehow dumped in front of your door. (P16, FG1)

The need for early planning referred not only to the discharge date itself but also to a jointly developed, everyday-relevant perspective. Several participants emphasized how important it was for them to know what would happen concretely after leaving the clinic:

And then also, okay, how does it continue? What happens then? I mean, the crisis is eventually over, then I leave the clinic. What do I do then? So that you already know, okay, at this time the discharge will happen, and then we have this and that planned. (I 13)

Joint conversations with clinicians and social workers were described as helpful, especially when social and everyday situations, support needs, and next steps were discussed:

Yes, so the first conversations were such that a regular appointment was scheduled, in which a social worker also participated, and yes, we discussed: What is the situation? What is the family situation? How should things proceed after leaving the clinic? And I found that very helpful, also to get support from the social worker, so to speak. Also when they helped to contact authorities, what to do next, to discuss perspectives after the episode. And I found that very helpful. (I 11)

A lack of coordination between inpatient facilities and outpatient support structures was experienced as a problem in several accounts, affecting both the well-being of next of kin and the person in treatment:

There are these recurring cycles of hospitalization when things aren’t well prepared. And outside, there’s actually a system ready to support this person, but they don’t use it, and they don’t take advantage of it. And crisis after crisis is reproduced again. (P16, FG1)

## Discussion

This participatory qualitative study aimed to explore what constitutes successful participation of next of kin in psychiatric settings from the perspective of next of kin themselves. Three themes were developed: [1] the need for a *network-conscious clinical culture* [2], different foci of participation (person-in-treatment-focused, relationship-focused, and next-of-kin-focused), and [3] changing needs over time. Taken together, the findings suggest that participants experienced meaningful participation as depending less on isolated interventions and more on a psychiatric culture characterized by relational, temporal, and structural sensitivity.

### From formal involvement to lived culture

Participants’ emphasis on a network-oriented clinical culture highlights the need for a broader cultural transformation. Rather than calling for specific interventions, they advocated a fundamental shift in professional attitudes namely, recognizing next of kin as legitimate and knowledgeable partners within a social network. This perspective aligns with recovery-oriented frameworks and social models of mental health, both of which stress relational embeddedness and shared responsibility [11, 35]. It also resonates with the normative commitments articulated in the 2006 Convention of the United Nations on the Rights of Persons with Disabilities, which emphasizes participation, autonomy, and the importance of supportive environments [12].

Our data indicate that participants perceived formal structures for involvement as insufficient when they were not embedded in the “lived culture” of the clinic.

Empirical findings indicate substantial variation in caregiver involvement across institutions reflecting differences in clinical cultures. A survey of persons in treatment, next of kin and hospital psychiatrists reported that psychiatrists had contact with caregivers in only about one third of inpatient cases (33.6%), with considerable variation between hospitals [36]. The most frequently cited reason for non-involvement was the perception that involvement was unnecessary (over 50%). Prior training in family work was associated with more positive attitudes and more frequent engagement.

Implementation research has identified several organizational facilitators, including a whole-ward approach, designated roles, standardized routines, supervision, and multidisciplinary collaboration (e.g [22]). Crucially, as Eassom and colleagues (2014) [13] argue, top-down endorsement or training of a limited number of staff members is insufficient; a whole-team and whole-organization approach is required. This underscores the need for cultural transformation rather than isolated guideline implementation. This aligns with Dörner and colleagues calling for a radical shift in perspective: clinicians should assume responsibility not only for the person in treatment but for the entire “social field” [37].

Participants’ accounts of feeling ignored, dismissed, or blamed for the illness are consistent with international evidence. Studies synthesizing family perspectives (e.g [20]). identify lack of recognition and respect as major barriers. Similarly, Wilkinson and McAndrew (2008) [18] describe carers’ experiences of exclusion during inpatient treatment, marked by feelings of powerlessness and invisibility. These experiences point not merely to communication deficits, but to deeper underlying hindering assumptions.

The theme of “power sharing” adds a critical dimension to this discussion. Participants did not describe empowerment as something that can be conferred upon them; rather, it was seen as contingent on professionals’ willingness to relativize their epistemic authority and to acknowledge uncertainty, emotional strain, and personal limits. This challenges traditional hierarchies in psychiatric settings and invites reflection on epistemic justice in clinical encounters. Importantly, such transparency was not experienced as undermining professional expertise but as fostering trust and reducing stigma. From the participants’ perspective, power sharing can be understood as a relational practice that may facilitate genuinely collaborative triadic encounters (clinician–person in treatment–next of kin).

From a conceptual perspective, these findings resonate with approaches emphasizing epistemic humility [38] and epistemic justice in healthcare [39], which call for recognition of different forms of knowledge and attention to whose perspectives are legitimized in clinical encounters. Importantly, power sharing should not be equated with unrestricted clinician self-disclosure. Rather, it refers to a relational stance characterized by openness, reflexivity, and recognition of uncertainty while maintaining appropriate professional boundaries.

### Differentiating the aims of participation

A second key contribution of this study lies in differentiating the objectives underlying next-of-kin participation. While the literature sometimes treats next of kin participation as a relatively uniform intervention category (e.g. psychoeducation), our findings suggest at least three analytically distinct yet overlapping foci.

First, person-in-treatment–focused interventions aim to enhance next of kin’s capacity to support the individual in treatment in everyday life. Requests for information, behavioral strategies, and accessible explanations were particularly prominent in the early phases. This aligns with quantitative evidence that psychoeducational approaches can improve coping and reduce caregiver burden [4, 8]. Our findings, however, add nuance by highlighting the importance of tone and framing: explanations were experienced as especially helpful when they were destigmatizing and normalizing, rather than merely technical. These insights also resonate with critical analyses cautioning against unreflective shifts of responsibility onto next of kin (e.g [40]). Previous studies have noted a tendency within psychiatric services to transfer responsibility onto carers without providing adequate support (e.g [41]). This relates to broader discussions of responsibilization and refamilization, in which increased family involvement may risk shifting care work from services to families without adequately acknowledging the resources, limits, and needs of next of kin. Policy analyses further suggest that increasing family involvement may sometimes reflect cost-containment strategies rather than genuine empowerment (e.g [42]).

Second, relationship-focused formats such as triadic or network conversations were valued as structured spaces for dialogue that are often unavailable in everyday family life, particularly during acute crises. Participants described these encounters as fostering mutual understanding, mediating conflicts, and sustaining connection. From this perspective, triadic and network conversations may provide a structured space in which differing perspectives and expectations of persons in treatment, next of kin, and clinicians can be openly negotiated, rather than assuming consensus from the outset. At the same time, several participants also highlighted that triadic conversations could be challenging, particularly when sensitive issues were difficult to address openly in the presence of the person in treatment. The findings suggest that triadic formats require careful facilitation and may entail learning processes for all parties involved. This aligns with Førde and colleagues (2016) [17], who emphasize that next of kin understand involvement as encompassing relational maintenance, dialogue, and recognition of their experiential knowledge not merely participation in decision-making. Such a broader conceptualization of participation supports approaches like network meetings or trialog, which seek to avoid instrumentalizing relatives and instead promote autonomy within the personal network.

Third, kin-focused interventions can directly address the well-being of next of kin themselves. Participants articulated a clear need for spaces in which their own feelings and experiences can be expressed without judgment. The desire for peer exchange and facilitated groups further suggests that participants valued opportunities for normalization and collective meaning-making, which they experienced as potentially reducing feelings of isolation. Importantly, such forms of support do not necessarily require access to confidential information or formal involvement in the treatment of the person in treatment. This distinction may be particularly relevant in situations where consent for involvement cannot be obtained or where the wishes of next of kin and the person in treatment diverge.

Taken together, these differentiated foci suggest that many participants understood participation as extending beyond information transfer or shared decision-making alone. Considering these three dimensions may help serviced reflect on how different forms of support can address the needs of the person in treatment, the relationship, and the next of kin as individuals.

### Needs may change over time

A third central finding concerns the dynamic nature of needs. Participants described distinct phases (shock, prolonged endurance, and transition periods) each associated with specific expectations. Early stages were often characterized by disorientation and existential anxiety, accompanied by a strong desire for proactive outreach, basic orientation, and emotional containment. Later, as next of kin “developed role(s)“, informational needs sometimes decreased, while expectations regarding collaboration and long-term support became more differentiated.

The characterization of next-of-kin involvement as a “prolonged endurance run” highlights the cumulative burden that can build up over many years. The transition from inpatient to outpatient care was repeatedly identified as a particularly vulnerable phase, often characterized by insufficient discharge planning and limited coordination. When discharges occurred abruptly and without clear agreements, next of kin experienced them as destabilizing for themselves as well as for the person in treatment. Collaborative discharge planning and continuity-of-care models (e.g., home treatment or integrated care pathways) therefore appear essential (e.g [3, 43]). 

These findings indicate that participants experienced participation as a longitudinal process that adapts to shifting circumstances rather than a one-time event (e.g., a family meeting).

Participants’ accounts suggest that early-phase low-threshold information, mid-phase endurance support, and structured, collaborative discharge management were experienced as different but complementary components of a temporally sensitive approach to participation.

### Strengths and limitations

A major strength of this study lies in its participatory design. The inclusion of researchers with experiential knowledge and collaboration with self-advocacy organizations enriched interpretation and helped avoid tokenistic involvement. The use of reflexive thematic analysis allowed for in-depth exploration of meaning-making processes.

As part of the reflexive process, we considered which perspectives were present and absent within the research team. While the team included researchers with diverse professional and experiential backgrounds, the perspectives of parents as next of kin were not directly represented in the analytical process, and lived experience of psychiatric care was less strongly represented, although both perspectives were represented in the steering board. This may have influenced data interpretation despite collaborative and reflexive analysis.

Moreover, several limitations must be acknowledged. Participants were recruited in part through self-help and next-of-kin organizations and a considerable proportion of participants were involved in such organizations (24/30). Consequentely, the sample may have been particularly engaged in or articulate about issues of participation. This may also have shaped the perspectives represented in the data, particularly regarding participants’ familiarity with mental health care structures and their ability to formulate broader systemic critiques. However, the recruitment strategy also included outreach via clinical staff to visitors of hospitalized patients, thereby enhancing diversity beyond members of next-of-kin organizations alone. All but one participant had experience as a next of kin in inpatient care. Most also reported experience with other treatment settings, particularly outpatient care, as well as day clinics and home treatment. However, as the sample was largely characterized by inpatient care and acute or severe mental health crises, the findings are likely to be most relevant to adult psychiatric services in similar contexts. The study design does not permit conclusions about setting-specific differences, and next-of-kin participation may differ in other areas of psychiatric care, such as outpatient settings. In addition, participation required sufficient German proficiency, which may have excluded individuals with limited German language skills whose experiences and cultural expectations regarding next-of-kin participation may differ.

Future research should therefore systematically analyze relatives’ experiences across different treatment contexts and include culturally and linguistically diverse populations. Finally, as the study was conducted within the German psychiatric system, the transferability of the findings to other health care contexts requires careful consideration. Furthermore, this study reflects only the perspectives of next of kin. The perspectives of persons in treatment and clinicians were not included, and future research should examine how participation is negotiated within the triadic relationship while balancing autonomy, confidentiality, safety, and the preferences of the person in treatment.

### Implications for practice

Several practical implications can be derived. First, fostering a network-conscious clinical culture requires organizational commitment beyond individual goodwill. This may include systematic early identification of key next of kin, routine offers of contact (subject to consent of the person in treatment), and clearly visible access points (e.g. designated consultation hours or next-of-kin liaison roles). However, as participants emphasized, such measures are only effective if staff are informed, trained, and supported to embody a relational stance.

Second, professional education might integrate reflective components addressing power, stigma, and epistemic hierarchies. Encouraging clinicians to acknowledge uncertainty and relational complexity may contribute to destigmatization and trust.

Third, participation should be conceptualized as multi-layered: interventions should target not only the person in psychiatric treatment but also relational dynamics and the wellbeing of next of kin. Integrating peer support and systematically informing families about self-help structures may strengthen resilience.

Finally, policy frameworks should recognize that next-of-kin participation is not solely an ethical imperative as articulated, for example, in the United Nations Convention on the Rights of Persons with Disabilities but also a structural requirement for sustainable care. Without adequate coordination across inpatient and outpatient sectors, the burden is often displaced onto next of kin. 

## Supplementary Information

Below is the link to the electronic supplementary material.

Supplementary Material 1: Interview Guide, in German, pdf-format.

## Data Availability

The datasets used and/or analysed during the current study are available from the corresponding author on reasonable request in the original language (German).
