# Supplementary material for: “It has to be part of the clinic’s lived culture”- perspectives of next of kin on meaningful participation in psychiatric care: a participatory thematic analysis
Source: BMC Psychiatry. 2026 Aug 26;26:675. doi: 10.1186/s12888-026-08561-5 (PMC13520185; doi:10.1186/s12888-026-08561-5)
Supplement: Supplementary file 1 [file 12888_2026_8561_MOESM1_ESM.docx]

| **Key Question** | **Possible Follow-up Questions** | **Probing and Guiding Questions** |
| --- | --- | --- |
| **Introduction** | |  |
| Do you have any questions before we begin? It’s great to have you here. Could you tell me what led you to participate in this interview today? | |  |
| **Subjective (Role) Experiences and Practice of Participation** | | - Would you like to tell me a bit more about that? - Do you have an example for me? - And then? What happened next? - How was it with…? - How was that for you? |
| How have you experienced the psychiatric care system as a next of kin? Is there a particular situation or experience you would like to share? | - What was discussed with you? - What was not discussed? - How did you feel about it? - What do you think clinicians expected from you as next of kin? - What role did you take on? - How did your relationship with your relative change during treatment? - If appropriate: What role could you have played? |  |
| **Structural and Personal Barriers** | |  |
| To what extent did you encounter challenges regarding your participation in the treatment? | - What kind of difficulties were these? - If appropriate: Why do you think practitioners involved you so little? - To what extent did you observe reservations or concerns in your relative? - Did you yourself have concerns about being involved? / What are your concerns about being more involved? - What role do time and financial factors play? |  |
| **Needs and Requirements Regarding the Participation of Next of Kin** | |  |
| In what ways would you ideally like to be involved in the treatment? | - What would have been helpful for you? - What would you have needed? - What is required for that? (clinical structures, financial structures, personal resources) - What should not happen? - What do you think your affected relative would want (regarding involvement)? - Have your expectations and needs changed over time? |  |
| **Significance and Value of Next-of-Kin Involvement in Treatment** | |  |
| In which situations do you think the involvement of relatives during treatment is important? | - For what purpose? For whom? - In which situations is it not important? |  |
| **Conclusion** | |  |
| Before we finish, is there anything important to you that we haven’t talked about yet? | |  |
